# Transactions of the Chicago Society of Physicians and Surgeons, at the Meeting June 9th, 1873

**Published:** 1873-07

**Authors:** P. S. Hayes


### Chicago Medical Journal.

A MONTHLY RECORD OF

*Medicine, Surgery and the Collateral Sciences.*

EDITED BY J. ADAMS ALLEN, M.D., LL.D.; AND WALTER HAY, M.D.

VOL. XXX. — JULY, 1873. — No. 7.

### Original Communications.

ARTICLE I.—*Transactions of the Chicago Society of Physicians and Surgeons, at the Meeting June 9th,* 1873. Reported by P. S. HAYES, M.D.

Paper on the Physiological Relations of Alcohol, by WALTER HAY, M.D. Read by invitation.

Cases, reported by W. C. LYMAN, M.D.

GENTLEMEN :—The present age has been termed that of skepticism. Within the domain of the physical sciences, skepticism and investigation are the parents of knowledge. He who doubts has awakened to the possibility of progress; he who blindly accepts dogmata sleeps under the tomb of tradition.

An occasional review of the principles of our professional faith may not be altogether unprofitable, if it results in nothing more than in the exercise of greater circumspection in their adoption and establishment.

The iconoclast, who tears down from their niches the idols of a false faith, may be the prophet of a new avatar.

The confusion, apparent in the minds of moralists, political economists, and physicians, regarding the influence of alcohol upon the human system, suggests the vagueness and uncertainty of the data upon which their opinions have been based, and indicates the possibility of deducing sounder conclusions, by elucidating its true physiological relations.

We are told by therapeutical authorities, that alcohol is a stimulant; lexicographers tell us that a stimulant in medicine is an article which produces a quickly diffused and transient increase of the vital energy and strength of the heart and arteries.

The dogma, that alcohol is stimulant, has been long considered a principle in therapeutics; let us examine upon what physiological foundations the principle rests; let us investigate its three-fold relations to the three essentially vital physiological functions, circulation, respiration, innervation.

The effects of alcohol upon the circulatory system are first made visible by an increased afflux of blood to that portion of the integuments of the face constituting the vascular area supplied by the ultimate ramifications of the ophthalmic artery, indicated by the flush of heightened color upon the cheek. The ophthalmic artery being the first branch of the carotid after its passage through the carotid foramen into the cranial cavity, the condition of its circulation will afford a very fair index of that of the entire encephalic circulatory system. Coincidently with these changes, occurs acceleration of the heart's action, indicated by increased frequency of the radial pulse. There is at the same time dilatation of the arteries and arterial capillaries, marked in the first case by augmentation in the number of red corpuscles present in the capillaries of the face, and in the second by the enlargement of the radial pulse.

The dilatation of the arteries of the central nervous system is manifested by symptoms to be considered under their appropriate head.

If the exhibition of the drug be continued, the effects just mentioned are intensified, the flush in the face deepens and extends more widely as new vascular areas are involved; the vessels of the conjunctivæ are engorged, and the swollen condition of the veins testifies to the dilatation of both the capillaries and trunks of the venous system likewise; pulsation diminishes in rapidity but not in volume, until the heart's action might even cease.

Upon the respiratory system the drug produces effects no less marked; first, an acceleration both of inspiration and of expiration appears coincidently with the increased rapidity of the heart's action and the dilatation of the capillaries already referred to; notwithstanding which, evidences of imperfect oxydation rapidly accumulate in the deepening color of the countenance and the turgidity of the venous system.

But the effects of alcohol upon the nervous system constitute the most reliable data from which conclusions regarding its true physiological relations may be deduced; while these are indissolubly correlated with the modifications of circulation and respiration already described, they, nevertheless, retain sufficient individuality to admit their identification.

The first of these to which I will direct your attention, is a modification of sensibility, a sense of heat or burning in the stomach and in the face. Whether this be due to a direct modification of sensory innervation, or to an indirect change resulting from the increased amount of oxygen carried to the terminations of the sensory nerves by the red corpuscles of the blood, has not yet been determined. Next, is a diminution, a quasi paralysis, of vaso-motor activity, manifested in the dilatation of the arteries already indicated. There is, moreover, impairment of the functional activity of the cerebral hemispheres, indicated by diminution in the energy of the will in dominating and controlling the activity of subordinate ganglia, resulting in insubordination of the emotional centres, and the centres of special sensation, whose impressions become confused, inaccurate and unreliable; the sensory and motor ganglia of the mesocephale respond reluctantly or abnormally to normal impressions, whether centripetal or centrifugal. Common sensation is impaired, muscular motions become irregular and incoördinate, until innervation generally is reduced to its lowest possible expression; the subject, by the abolition successively of the functional energy of the superior ganglia of the nervous system, is reduced gradually, through all the lower phases of animal life, to a merely vegetative existence.

The symptoms induced by alcohol having been considered as much in detail as the limits of this paper will permit, their physiological significance may next be discussed.

The first of these, increased afflux of blood to the arteries and capillaries, can only occur by dilatation of those vessels (farther shown by increased fullness of the radial pulse), and this, again, by relaxation of their muscular walls; now the calibre of blood vessels is regulated by the amount and degree of nervous irritability, hence dilatation of the calibre of blood vessels is simply another expression for vaso-motor paralysis or quasi paralysis, for withdrawal of nerve force. Hence it appears that the first effect of alcohol upon the circulatory system, with its correlative expression through the nervous system, is *diminished* rather than *increased* vital energy.

But the pulsations of the heart are at the same time increased in number, and this fact constitutes the corner stone of the stimulant theory of alcohol.

This corner stone, however, will crumble under the hammer of physiological criticism. It would certainly be a physiological paradox, that an agent which can induce a condition of diminished vital energy in one portion of the nervous system, should occasion the opposite state in another; modern physiology has explained the paradox by demonstrating the inhibitory action of the pneumogastric nerve upon the heart, and shown that whatever interrupts its influence upon that organ occasions in it increased rapidity of action. This necessitates the admission of reciprocity of action in the heart and arteries, without which these phenomena and their modifications would be inexplicable.

The effects upon the circulation in the face are identical in character with, although of course infinitely less conspicuous than, those induced by section of the cervical sympathetic.

Coincidently with these again is temporarily increased rapidity of respiration, the accelerated arterial necessarily involves greater rapidity in the venous circulation also, and an increased afflux of blood to the lungs, whose vessels are moreover less able to resist it, the result of which is the necessity for more rapid oxydation through the respiratory surface, evidence of disturbed co-ordination between respiration and circulation, the direct result of partial arrest in the innervation of the pneumogastric nerve, as determined by actual experiment to be temporary acceleration with consecutive retardation of respiratory movements, with engorgement of pulmonary vessels. To these must be added the evidences of diminished exhalation of carbonic acid from the respiratory surface, amounting, as shown experimentally, to twenty-five per cent., after the ingestion of even a small quantity of alcohol.

But it has been shown that there was a hyperæmic condition, so to speak, of the ophthalmic artery, and inferentially of the brain. Now, hyperæmia of the cortical portion of the hemispheres of the brain is the physiological equivalent of the metaphysical condition which is termed delirium, and this condition is typified in the intellectual aberrations of the alcoholized subject. There are probably very few left who recognize in the incoherent ravings of a maniac any evidences of *increased vital energy*, but rather of energy perverted and degraded.

Or shall we look for such evidence in the disorderly manifestations of emotional activity which has become relatively conspicuous by reason of the degradation of the higher faculties, which in a normal condition regulate and control their energy?

Or, again, do we find it in the more or less complete suspension of functional activity in the organs of special sensation as the medulla oblongata becomes involved in this hyperæmic condition and their nervous centres are successively implicated? The dimmed eye, the deafened ear, the stammering tongue, all indicate paralysis, and to these are added, as the hyperæmia gradually suspends the functional energy of cerebellum and spinal cord, vertigo, anæsthesia, muscular incoördination, and paralysis.

From the first symptom to the last, paralysis is the essential lesion in every phase of acute alcoholism. Whether we consider the dilatation of arteries, the increased blood supply, the escape of the heart from its normal control, diminished volitional energy, disordered intellection, irrepressible emotional activity, diminished special sensibility, suppression of pulmonary exhalation, and glandular secretion, loss of common sensation and of motor power, separately or together, it will be perceived that each one of them points back to its ultimate factor, vaso-motor paralysis, diminished nerve-power.

The facts adduced thus far, although they comprise only the salient points in the subject of the physiological relations of alcohol, and sufficient only to enable me to sketch the bare outlines of its history, will, I think, indicate clearly that, so far from increasing vital energy and strength of the heart and arteries, alcohol diminishes both, and thus fails to fulfill the definition of a stimulant.

The object of this paper is not to favor a blind and unreasoning prejudice against the use of this drug, either intra- or extra-professionally, but by pointing out its real effects to suggest the conditions in which it may be used most beneficially in the alleviation of the ills of human life, and thus be retained within the category to which it originally belonged, of blessings to humanity, instead of being condemned to that into which physiological blindness has permitted it to be relegated, of the curses.

The question whether the drug be or be not a stimulant, may be said to be simply a war of words, but words are symbols of ideas, and men are governed by symbols rather than by ideas, hence the acceptation of a false symbol involves subordination to its correlative false idea.

A correct appreciation of the true physiological relations of alcohol will result, it is to be hoped, in the substitution of a more strictly scientific therapeutical application of the drug for the almost purely empirical method at present in use by many, even in the front ranks of our profession; will prevent in the future the administration of this drug to relieve chronic engorgement and hypertrophy of the liver by one distinguished practitioner, or to sustain the failing energy of a fatty heart by another.

Much of the demoralization which has resulted from the abuse of this drug extra-professionally, has its source in professional ignorance, and while we are not willing to be held accountable for any considerable share of human misery, we are bound to investigate for ourselves a subject, upon whose correct appreciation by physicians such important issues depend.

Gentlemen, I trust that the shortness of time at my command for reducing these ideas to a presentable shape, and the necessity for condensing their expression within the limits of a brief paper, will be a sufficient apology for the cursory manner in which I have treated a subject which admits of and deserves the utmost elaboration.

Permit me, in closing, to thank you for the honor which you have done me in your invitation, and the courtesy which you have shown me in your kind attention.

After the reading of the paper a general discussion ensued, in which most of the members participated, and in which Dr. Hay made special application of the principles set forth in the paper. After this, Dr. Lyman read a report of the following cases which occurred in his practice. He was induced to present them to the society, more from an idea of their possible novelty than any special lesson that they might teach.

---

Case 1. Mrs. S., a healthy young woman about 28 years of age; nine years married; had never before supposed herself pregnant; applied to me to attend her in her approaching confinement, in September last, stating that she expected her labor to occur by the middle of October. October came and nothing was heard of the case till near the end of December, when the husband called upon me in a state of anxiety, which may be considered perfectly natural when it is understood that he left home on January 15th, previous, and his wife had written him of her (and his) good fortune, while absent; and he found his wife, on his return, in that interesting state (apparently) that "ladies delight to be, who love their lords."

To quiet his mind, I explained how sometimes a case of the kind went on to a period far beyond the average 280 days, and this was probably one of them. This satisfied him for a time, but shortly he returned, and I fairly thought that the pregnancy must have commenced on his return, June 15, 1872, which would render her labor due March 15, 1873. This date came, and they consented to an examination. I found the abdomen enlarged to the extent of an eight months pregnancy, the prominence being slightly to the left side. There were movements that were to all appearance so like the movements of the fœtus in utero, that they well might deceive both patient and physician, but there was no sound of the fœtal heart. Examination per vaginam showed the womb in nowise enlarged, greatly retroverted, but presenting no evidence of any other disease or disorder. Of extra-uterine pregnancy, ovarian cyst, or solid tumor, there were none. There were only the prominent abdomen and apparent fœtal movements to account for. These have gradually passed away since she has been assured of her true condition. I can only conclude that the profound mental impression that she was to bear a child, had its effect upon the economy of nature, so as to develop this growth of and produce muscular movements of the abdominal wall, such as might attend real pregnancy.

Case 2. I desire to relate the following case more to put it on record than anything else. Miss A., a young lady 27 years of age, consulted me respecting the propriety of her marrying. Having never menstruated, she suspected all was not right with her. She was of full height, osseous and muscular; system well developed; face, figure and voice essentially feminine.

Examination of the genitals showed a rudimentary development of mons veneris, clitoris, labiæ, and fourchette, the extension formed of the perinæum to the pubic arch being thick, firm and fibrous. Directly below the pubis was a little pocket lined by mucous membrane, about one-third of an inch in diameter, and about three-quarters of an inch in depth, in the upper wall of which the urethra opened with papilla-like eminence—this opening was a rudimentary vagina. For fourteen years she has had a monthly return of the symptoms that ordinarily attend menstruation, and was frequently conscious of sexual desire. Hence I conclude that the ovaries are developed and possibly the uterus, for I made no examination per rectum, (the purpose of the consultation being subserved by the answer that she could not marry), and the external genitals wholly rudimentary.

Case 3. In July, 1872, first saw Wm. Thorpe, stone-cutter, age 29, of slight build, average intelligence, native of Scotland. During the noon hour had laid down beside one of the masses of stone on which he had been at work. It fell over upon him, and he was taken up wholly insensible. An hour later he appeared as follows: A condition of profound shock, with the usual coldness, weak pulse, sighing, respiration and tossing to and fro, right side of the face frightfully bruised, with the eye protruding, and a perpendicular fracture with depression of the outer table, two inches in length, and one inch behind the right ear.

It is useless to detail the treatment, that in four or five days was followed by a return of consciousness, attended by most severe pain in the head, which gradually diminished till at the end of sixty days he was free from it. The right eye remained largely prominent and the orbit full, the conjunctiva enormously œdematous, and excessively painful.

Two weeks from the reception of the injury, obscure fluctuation was observed over the globe of the eye, and an abscess found at the depth of one inch, from which two ounces of sero-purulent matter were discharged, together with a number of pieces of a white apparently fibrous mass, that proved to be portions of white brain tissue. Exploration of the abscess by Dr. Jackson, (who was present at this time), showed fracture of the superior orbital plate, with depression of its posterior part, pushing the eye permanently forward, and admitting the probe into the cranial cavity. A fracture extending directiy backward, horizontally, met the perpendicular fracture behind the ear, traversing the petrous portion of the temporal bone, doubtless in a straight line, and admitting the probe to the depth of four and one-half inches, far enough to meet the perpendicular portion of the fracture.

A slight degree of relief followed the evacuation of the abscess, and a serous discharge continued for about two weeks, when the external opening closed spontaneously. At the end of two months he was discharged, well, and at the end of three months resumed his work, stone-cutting.

The only unpleasant sequel of the case has been the occurrence of convulsions of epileptiform character about a week after he resumed work, induced by stooping at his work in the hot sun. Two drachms of bromide of potassium, given daily for three or four days, apparently controlled this symptom, for since that time (about nine months) it has not returned.

His present condition is noticeable from the fact that the right eye is half an inch in advance of the left; the sight only impaired by the want of accommodation with its fellow, caused by displacement only.

I believe this case to be chiefly remarkable for its recovery after a portion of the brain had been torn off, and expelled from the cranial cavity by occurrence of abscess.

Case 4. Claude McIntosh, age 24, of previous good health, of irregular habits, colored, born of healthy parents, family numerous, all healthy. First felt ill about the 1st of December last. Had occasional attacks of pain in the abdomen, for which, to use his own words, he "would take two or three drinks," and it readily passed away. This, together with habitual constipation, continued until about February 1, when he, for the first time, gave up his employment upon one of the sleeping car lines. From this time the attacks of severe pain occurred almost daily, often lasting two or three hours, when they would pass away, leaving him entirely comfortable. With these symptoms, a moderate appetite, tongue slightly furred, no febrile action, no undue fullness, tenderness, or hardness of the abdomen. The bowels were easily moved by moderate doses of any laxative medicine. He was several times quite freely purged, with apparent relief, but ultimately the pain always returned. About the middle of March, after a fortnight of comparative quiet he had a return of the pain; it often came on several times daily, and subsided without apparent reason. He was put on the steady use of opium till the pain was controlled, and when entirely quiet, an emulsion of two ounces of castor oil administered. This was followed by the evacuation of two or three dozen scybalæ of the size of a chestnut, and another smaller dose of oil evacuated a solid mass, three inches in length and one and three-fourth inches in diameter. I thought then the trouble was over, but in reality it had just begun. Great pain followed in the left iliac fossa, with extreme local tenderness and moderate swelling, the pulse for the first time became quickened and rose to 140. All the symptoms of acute peritonitis were rapidly developed. Opiates were largely given, beginning with three grains of morphine daily, and rapidly increasing it, as a tolerance of it became established, till he took eight grains of the sulphate daily. Enough was given without regard to quantity to control the pain and reduce the rapidity of cardiac action. When once fully impressed by the sedative influence of the opiate, the symptoms abated, and at the end of two weeks the bowels were again evacuated, moving freely with little pain, the tenderness, tympanitis, and prominence of the left iliac region, being reduced. Two or three dozen more little masses, and one large one, even larger than the other, were voided without the use of any purgative. With abated fever, swelling and pain, and returning appetite and sleep, I thought that the end had come, but the end was not yet. Ten days of apparent convalescence, during which he took daily fluid food, and had stools, were followed by a return of pain and abdominal tenderness. In anticipation of another large scybala, I ordered an enema of infusion of elm bark; it was followed by a copious stool, containing another mass larger than either voided before, being fully three inches long and two inches thick, shaped not unlike a potato, and quite as hard. Entire relief from pain followed, but in an hour another stool of nearly pure blood followed, then others, and before two hours had elapsed he was dead from hemorrhage.

Autopsy thirteen hours after death by Dr. Jackson and the writer.

Abdomen only opened. A general view of the interior of the abdomen showed the presence of general peritonitis, with adhesions existing between the convolutions, very little fluid in the cavity, the livid look of intense inflammation over the whole of the peritoneum, visceral and parietal. The omentum was shriveled to an inch of irregular fringe across the upper part of the abdomen. The whole surface of the peritoneal membrane and the omentum was covered by countless little masses from the size of a mustard seed to that of a grain of wheat. These were too solid to be the ordinary product of inflammation, and a specimen placed under the microscope showed it to be tubercular.

Examination of the small intestine showed nothing worthy of remark. The appendix was a shriveled, knotted cord, less than two inches long, the caput coli was largely distended, being nearly or quite four inches in diameter, doubtless caused by a narrowing that existed, commencing at the sigmoid flexure and extending upward seven or eight inches, and diminished in calibre so as to no more than admit the finger. All along this portion it was thickened, indurated and contracted by inflammation, involving the whole structure—not alone the peritoneum, which elsewhere seemed alone affected, but the muscular and mucous coats as well. It was from this part, doubtless by rupture of a vessel by distension, when the last mass was voided, that the fatal hemorrhage came.

I am inclined to think that the tubercular deposit was one of the later occurrences in this case, as miliary tubercle of the lungs has often produced symptoms of acute pneumonia, or, possibly, during the progress of an acute pneumonia this form of tubercle has been deposited.

I also think that the large masses expelled were the result of a long period of constipation and imperfect digestion, the large intestine becoming to a great degree sacculated, accommodating itself to the presence of the obstructions, and when they are suddenly, from any cause, removed, the newly exposed part readily taking on inflammation, or an already existing chronic inflammation suddenly taking the acute form.

